# Knowledge and practices on sustainability in nephrology in Brazil

**DOI:** 10.1590/2175-8239-JBN-2026-0110en

**Published:** 2026-11-09

**Authors:** Fabiana Baggio Nerbass, Nandressa D. Mendes Riso, Cinthia Kruger Sobral Vieira, André L Pimentel, Ana Flavia Moura, Luis Claudio Pinto, Fátima R. Veiga Tostes, Rafael B. Trancoso, Talita G. Salani, José A. Moura-Neto

**Affiliations:** 1 Sociedade Brasileira de Nefrologia, São Paulo, SP, Brazil.; 2 Fundação Pró-Rim, Joinville, SC, Brazil.; 3 Hospital Universitário do Oeste do Paraná, Cascavel, PR, Brazil.; 4 Clinefro Serviço de Nefrologia e Diálise, Porto Alegre, RS, Brazil.; 5 Associação Brasileira de Centros de Diálise Transplantes, Brasília, DF, Brazil.; 6 Escola Bahiana de Medicina e Saúde Pública, Salvador, BA, Brazil.; 7 Universidade Federal do Pará, Belém, PA, Brazil.; 8 Hospital São Vicente de Paulo, Rio de Janeiro, RJ, Brazil.; 9 Hospital Santa Rita, Vitória, ES, Brazil.

**Keywords:** Kidney Diseases, Renal Dialysis, Climate Change, Environment

## Abstract

**Introduction:**

Climate change has a bidirectional relationship with kidney diseases: environmental stressors may increase the incidence and progression of renal conditions, while dialysis therapy is among the treatment modalities with the highest environmental impact in healthcare. Despite the growing international debate on sustainable nephrology, this topic remains underexplored in the Brazilian context.

**Objective:**

To investigate the level of knowledge, perceptions, and practices related to environmental sustainability among nephrology professionals in Brazil and to identify barriers to the adoption of sustainable strategies in dialysis services.

**Methods:**

This cross-sectional study was conducted using an online questionnaire targeting nephrology professionals. The instrument assessed knowledge regarding climate change and kidney health, sustainability practices implemented in dialysis services, and institutional barriers to their adoption.

**Results:**

A total of 180 responses from professionals across 20 Brazilian states and the Federal District were analyzed, with a predominance of nephrologists and nurses. Most participants recognized the impact of climate change on kidney health; however, limited knowledge was observed regarding public policies and structured sustainability strategies. The most frequently reported practices involved waste management, whereas initiatives such as water reuse, renewable energy sources, and sustainable transportation strategies were less prevalent. The main barriers identified included financial constraints, lack of institutional incentives, and absence of strategic planning.

**Conclusion:**

These findings highlight gaps between perceptions, knowledge, and the implementation of sustainable practices in Brazilian nephrology, reinforcing the need for policies, professional training, and institutional strategies to promote environmentally sustainable kidney care.

## INTRODUCTION

Climate change poses a growing threat to human health and well-being. According to the World Health Organization, climate variability is already associated with humanitarian emergencies caused by heatwaves, wildfires, floods, tropical storms, and hurricanes, whose frequency and intensity have increased in recent decades^1^.

Kidney diseases and climate change have a bidirectional relationship, in which each can amplify the effects of the other^2^. Rising temperatures are associated with a higher incidence of nephrolithiasis, acute kidney injury, and the progression of chronic kidney disease^3^. On the other hand, the treatment of kidney disease, particularly through dialysis, has one of the highest environmental footprints among healthcare modalities due to the high consumption of energy, water, and disposable plastic materials^4^.

In recent years, nephrology has been increasingly recognized as a strategic field within the global health sustainability agenda. Given the current scenario, a substantial increase is expected in the number of individuals undergoing dialysis treatment and, consequently, in water and energy consumption, as well as carbon emissions from the sector in Brazil^5,6^. Comparative international analyses show that both the environmental impact and the organization of kidney care vary widely across countries, reflecting structural, economic, and regulatory differences in healthcare systems^7^.

In this context, global initiatives and position statements have emerged, and several nephrology societies have published articles and recommendations on sustainable nephrology and dialysis, reflecting the growing recognition of the environmental impact of kidney care^8,9,10^. The International Society of Nephrology launched the “GREEN-K” (Global Environmental Evolution in Nephrology and Kidney Care) initiative, which emphasizes the need to strengthen health promotion strategies, prevent and delay kidney disease progression, expand access to transplantation, and develop more environmentally sustainable dialysis therapies. The document also highlights that education and professional engagement are essential for the implementation of sustainable practices^2^.

In Brazil, this movement has advanced in recent years^11^. In 2023, the Brazilian Society of Nephrology (SBN) established the Sustainable Nephrology Committee, responsible for developing national recommendations aimed at incorporating sustainable practices into kidney care. These recommendations were subsequently presented and approved in 2025 at the 2nd Convention of the BSN Board and its Regional Chapters. The document consolidates ten strategic recommendations, with progressive implementation planned for the period from 2027 to 2030, representing an institutional milestone in the sustainability agenda of Brazilian nephrology^12^.

Additionally, sustainability has been increasingly incorporated into the scientific agenda of Brazilian nephrology. The Brazilian Congresses of Nephrology in 2022 and 2024 included this topic in their scientific programs. The 2024 edition, held in Salvador (BA), marked a milestone by measuring the event’s carbon footprint and offsetting 128 tons of CO_2_equivalent (tCO_2_e) through the Envira Amazonia Forest Conservation Project^13^.

Despite these advances, healthcare professionals’ knowledge regarding sustainability in nephrology remains limited. A survey including approximately one thousand professionals from 108 countries showed that fewer than 50% were aware of the effects of climate change on kidney health or of the environmental impact of kidney care, and only 26% reported working in organizations with sustainable initiatives^14^. Studies conducted in other countries indicate that these knowledge gaps and the heterogeneous implementation of sustainable practices in nephrology are not limited to low- and middle-income countries but are also observed in well-established healthcare systems such as those in Canada, European countries, and Japan^15,16,17^. There are currently no data assessing these issues within the nephrology community in Brazil.

Therefore, this study aimed to map the knowledge, attitudes, and practices of healthcare professionals working in nephrology regarding the interconnection between climate change and kidney health, as well as to identify sustainable organizational initiatives and practices.

## METHODS

### Study design

This was a cross-sectional study conducted using an online questionnaire developed by members of the Sustainable Nephrology Committee and the board of SBN The instrument was reviewed by other members of the Sustainable Nephrology Committee with expertise in sustainability in nephrology and survey methodology. Pilot testing was conducted with healthcare professionals from different backgrounds to assess clarity, comprehension, and content adequacy. This process constituted a qualitative assessment of the instrument, and no formal psychometric validation or quantitative evaluation of content or construct validity was performed.

The questionnaire was disseminated through multiple channels, including email invitations sent to members of the SBN and to the email addresses registered by dialysis units, as well as through institutional social media platforms and messaging application groups. No quotas or stratified sampling by professional category were established. Participation was voluntary and open to all healthcare professionals involved in nephrology care. Responses were collected between May and July 2025, and the full questionnaire is presented in the supplementary material.

### Population

The survey targeted healthcare professionals involved in the care of patients with kidney disease, including nephrologists,nurses, technicians, and other professionals in the field. No exclusion criteria were established regarding workplace setting or years of professional experience.

### Data collection

Participants completed the online questionnaire using Google Forms. The instrument included structured questions addressing the following topics: (1) sociodemographic and professional characteristics; (2) knowledge about climate change and its relationship with kidney health; (3) sustainable practices adopted in healthcare services; and (4) institutional barriers to the implementation of sustainable strategies (supplementary material).

### Ethics

The study was conducted in accordance with ethical principles governing research involving human subjects. No information allowing personal identification or identification of the workplace was collected. The study was approved by the Research Ethics Committee of Hospital Ernesto Dornelles (approval no. 7,461,891, dated March 25, 2025) and was registered under the Certificate of Presentation for Ethical Consideration (CAAE) no. 86807325.2.0000.5304.

## RESULTS

### Participant characteristics

A total of 180 respondents were included in the study. Participants represented 20 Brazilian states and the Federal District, with a predominance of respondents from the Southeast region (41%), followed by the South (37%), Northeast (9%), Central-West (7%), and North (5%).

Among participants, nephrologists predominated (51%), followed by nurses (28%) and other healthcare professionals (21%). Half of the respondents had more than 20 years of experience in nephrology, while 24% had between 11 and 20 years of experience and 26% had less than 10 years. Regarding the funding source of the dialysis unit where they worked, 67% reported that the care was funded exclusively or predominantly by the Brazilian Unified Health System (SUS).

### Perceptions of climate change and kidney health

Slightly more than half of the respondents (54%) reported a higher level of concern regarding environmental impact and climate change, whereas 46% reported low concern or a neutral position.

Regarding the relationship between climate change and the health of patients with kidney disease, 57% believed there is a strong impact, and 35% believed there is some impact, while 8% reported being neutral or perceiving little or no impact.

In terms of knowledge on the topic, 38% reported having little or no knowledge, 48% reported some knowledge, and 13% reported a high level of knowledge. Only 36% reported being aware of any public health policies addressing climate change.

### Sustainability practices in dialysis units

Table 1 presents three questions related to sustainability in the participants’ centers and clinics. Overall, most respondents reported the presence of sustainable initiatives, awareness programs, or consideration of environmental impact in decision-making, although a substantial proportion either lacked this information or did not report adopting such practices.

**Table 1:** Sustainability practices and perceptions in dialysis clinics.

Question regarding the dialysis unit	Yes	No	I don’t know
Are there any sustainable measures/initiatives?	64%	21%	15%
Is there any type of awareness or education on sustainability and the environment?	64%	27%	8%
Is environmental impact considered in decisions regarding resources, consumption, or transportation?	57%	28%	14%

The main practices related to waste, water, and energy management in dialysis centers are described in Table 2. Regarding waste management, the most frequently reported practices were proper waste disposal (79%), separation of recyclable waste (66%), and availability of recycling bins (62%).

**Table 2:** Practices adopted by dialysis centers.

Resource	Practices	%
Waste	Proper waste disposal	79
	Separation of recyclable waste	66
	Availability of recycling bins (plastic, glass, paper, organic waste)	62
	Reduction and responsible use of paper	58
	Measures to reduce waste generation	53
	Preference for products with recyclable packaging	28
	Preference for biodegradable materials, when available	23
	No measures adopted	3
	I do not have this information	5
Water	Use of municipal water supply	69
	Water-saving measures implemented	40
	Water-efficient faucets (flow-control valves)	34
	Reuse of reject water from water treatment for other purposes	31
	Additional osmotic treatment of reject water	18
	Use of groundwater	17
	Rainwater harvesting	7
	No measures adopted	3
	I do not have this information	8
Energy	Use of electricity supplied by a utility company	63
	Turning off electronic devices when not in use	57
	Use of energy-efficient lighting	56
	Attention to indoor climate and thermal comfort	45
	Use of energy-efficient equipment / other energy-saving measures	32
	Use of photovoltaic (solar) energy	30
	Use of motion sensors for lighting	28
	Other energy-saving measures	15
	Use of energy from renewable energy providers	11
	Transportation options with reduced environmental impact	3
	No measures adopted	3
	I do not have this information	6

Note – Percentages may not total 100% because respondents could select more than one option.

Regarding water use, most centers relied on the municipal water supply (69%) and implemented water-saving measures (40%), including the use of water-efficient faucets (34%) and reuse of discarded water (31%).

In terms of energy use, the most commonly adopted measures included turning off equipment when not in use (57%) and using energy-efficient lighting (56%). Less frequently adopted measures included solar energy use, renewable energy providers, and environmentally friendly transportation.

Additional actions reported by participants are described in the supplementary material.

### Sustainability, cost savings, and barriers to implementation

Regarding perceptions of financial return on sustainability investments, most participants indicated that measures aimed at reducing environmental impact and optimizing resource use result in cost savings, particularly in the long term (39%) and the medium term (28%). A smaller proportion believed that returns occur in the short term (7%) or that such investments do not generate financial returns (5%). Approximately 21% of respondents reported lacking information on this topic.

Participants identified financial constraints (68%) and lack of incentives from the Ministry of Health and governmental bodies (62%) as the main barriers to implementing measures to reduce environmental impact. Other relevant challenges included the absence of a strategic plan (39%), limited knowledge of the topic (32%), and lack of local support (27%). Secondary barriers included a shortage of human resources (13%), lack of adequate technology (20%), and other factors reported by 7% of respondents.

## DISCUSSION

The findings of this study reveal that, although there is increasing awareness of the relationship between climate change, sustainability, and kidney health, important gaps persist in knowledge, the implementation of practices, and institutional support within Brazilian dialysis units.

Similar results have been observed in a survey conducted in Canada, where nephrology professionals also demonstrated significant knowledge gaps regarding environmentally sustainable kidney care strategies, particularly in areas related to institutional policies, energy use, and water management, even within a more structured healthcare system^18^.

Our sample included professionals from different regions of the country, with a predominance of participants from the Southeast and South. Most respondents were nephrologists wiTabela 1Tabela 1th extensive experience in the field. In addition, the majority worked in units predominantly funded by the SUS, a context that may directly influence the capacity to invest in and implement sustainable practices.

Half of the respondents had more than 20 years of experience in nephrology. This characteristic may have influenced the findings, as professionals at different career stages may differ in their familiarity with sustainability concepts, institutional responsibilities, and perceptions of environmental challenges. Although more experienced professionals may provide valuable insights into long-term changes in kidney care, younger professionals may have been exposed to sustainability topics more frequently during their education and training.

### Perceptions of climate change and kidney health

The results indicate that nephrology professionals recognize the impact of climate change on the health of patients with kidney disease, although this recognition does not yet translate into in-depth knowledge of the topic. Indeed, the scientific literature has shown that, despite the rapid growth in publications addressing the impact of climate on nephrology, the integration of this knowledge into clinical practice and health policy planning remains limited. Most professionals acknowledge the issue, but few have specific training or access to adapted protocols for mitigation and adaptation of kidney care in the context of climate change^2,19^.

Two additional findings deserve particular attention. First, nearly all respondents (97.8%) indicated that more information on sustainable nephrology and resource optimization strategies is needed. This finding highlights a substantial unmet demand for education and training in this area among nephrology professionals. Second, fewer than half of the participants (44.4%) reported being familiar with the terms ‘Green Nephrology’ or ‘Sustainable Nephrology,’ despite the establishment of the Brazilian Society of Nephrology’s Sustainable Nephrology Committee in 2023. Together, these findings suggest that, although sustainability has gained visibility within scientific and institutional agendas, awareness and dissemination efforts have not yet reached a large proportion of the nephrology community in Brazil.

### Sustainability practices in dialysis units

Most participants reported the presence of sustainable initiatives, educational actions, and consideration of environmental impact in decision-making within their units, although a substantial proportion still lacks awareness of or does not adopt such practices. This heterogeneity is consistent with qualitative and quantitative studies showing that engagement in sustainable practices varies widely across different contexts and professional profiles, influenced by factors such as the level of knowledge, individual values, practical barriers, and organizational characteristics^20,21^. Specifically in nephrology, international organizations promoting global actions for sustainability, education, and collaboration recognize that implementation remains heterogeneous and that many professionals are still not engaged or informed about sustainability^2,8,16^.

### Waste management

The findings of this study show that waste management practices are among the most established sustainability actions in dialysis units. The high frequency of reports of proper waste disposal and recycling suggests that centers have already incorporated basic waste management procedures, which is particularly relevant given the large volume of disposable materials used in dialysis.

Initiatives promoting responsible paper use reinforce this trend, although more advanced practices—such as prioritizing recyclable packaging or using biodegradable materials—remain uncommon. This heterogeneity aligns with the national context, where overall recycling rates remain low (8.3% in 2023)^22^ and implementation depends on local infrastructure, logistics, and regulatory incentives.

An initiative reported by dialysis units in Brazil involves the sale of high-density polyethylene (HDPE) containers and cardboard boxes used for packaging supplies, with proceeds allocated to purchasing basic food baskets for socially vulnerable patients^23^.

### Water management

Regarding water use, the results indicate that, although supply is broadly ensured, the adoption of strategies aimed at conservation and efficient use remains limited from the professionals’ perspective. The predominance of municipal water supply (69%) reflects reliance on a continuous and safe source, essential for dialysis water treatment systems.

However, water-saving measures were reported by only 40% of respondents, and specific practices such as water-efficient faucets or reuse of reject water—reported by approximately one-third of participants—suggest substantial room for expanding sustainable initiatives in water management.

In Brazil, although RDC No. 11/2014 establishes standards for dialysis services and the quality of water used in kidney replacement therapy, there is limited national regulation specifically addressing the reuse of reverse osmosis reject water for non-potable purposes. Consequently, the implementation of water reuse strategies remains dependent on local infrastructure, institutional policies, and operational protocols, representing an additional challenge for the broader adoption of sustainable water management practices in dialysis units.

Each hemodialysis session consumes, on average, approximately 500 liters of water, and about two-thirds of this volume are rejected by reverse osmosis systems and discharged into the sewage system^8^. In this context, circular management strategies—including reduction, reuse, and recycling of water—have been recommended in international publications, although their implementation remains limited worldwide^24,25^.

### Energy management

The findings show that basic energy efficiency measures are more common than structural strategies involving a transition to renewable sources. Most respondents reported simple practices, such as turning off equipment when not in use and using energy-efficient lighting, indicating that easily implementable actions are already part of routine practice in many units.

In contrast, practices requiring greater financial investment or infrastructure changes remain less frequent: 30% reported using photovoltaic energy, 11% reported access to renewable energy providers, and 28% reported using motion sensors. International studies describe a similar pattern.

In a survey conducted in Australia and New Zealand, approximately 44% of dialysis units used motion sensors for lighting, whereas only 14% reported using renewable energy, either through solar panels or green energy contracts^25^. Several barriers have been identified to explain this low adoption, including financial constraints, lack of institutional incentives, absence of specific public policies, infrastructure limitations, lack of technical knowledge, and low prioritization of environmental issues in administrative decision-making^9,25^. Thus, there is significant room to expand the use of structural strategies and renewable energy sources in dialysis units. Overcoming institutional, economic, and educational barriers will be essential to advancing energy sustainability and reducing the environmental footprint of kidney care. A recent scoping review evaluating sustainable interventions in kidney care reinforces that, despite growing global interest in the topic, the adoption of sustainable practices remains fragmented, concentrated mainly in hemodialysis, and limited by the absence of standardized metrics, institutional governance, and integration into health policies^26^.

### Sustainability, cost savings, and barriers to implementation

Participants’ perceptions regarding the relationship between sustainability and cost savings indicate that most recognize the potential financial return of such initiatives, particularly over longer time horizons. For most respondents, economic benefits become more evident in the medium to long term, while approximately one-fifth reported having insufficient information to form an opinion.

Financial constraints were identified as the main barrier (68%), followed by a lack of incentives from the Ministry of Health and governmental bodies (62%). In addition, internal organizational factors represent relevant obstacles.

The absence of a strategic sustainability plan (39%), limited technical knowledge (32%), and perceived lack of local support (27%) suggest that training, awareness, and governance processes may play a crucial role in strengthening the environmental agenda within units.

International experiences indicate that the implementation of environmental performance indicators, financial support for technological upgrades, and public policies focused on sustainability are key determinants for increasing both impact and adherence to sustainable practices^9,27^.

European studies suggest that the presence of formal organizational structures, such as dedicated sustainability teams (“Green Teams”), is associated with greater implementation of environmental practices in dialysis units, highlighting the importance of institutional engagement in translating awareness into concrete actions^16^.

### Strengths and limitations

The main limitations of this study are the use of convenience sampling and voluntary participation, which introduce the potential for selection, non-response, and volunteer biases. Consequently, professionals with greater interest in environmental sustainability may have been more likely to participate, potentially leading to an overestimation of the level of knowledge, engagement, and adoption of sustainable practices. Additionally, because no quotas or stratified sampling by professional category were used, the observed distribution among professional groups (51% nephrologists, 28% nurses, and 21% other professionals) likely reflects differential access to and engagement with the dissemination channels used, rather than the actual composition of the nephrology workforce in Brazil. In addition, because the survey was disseminated through multiple open channels, it was not possible to determine the total number of healthcare professionals reached or to calculate the response rate, limiting the assessment of sample representativeness. The underrepresentation of the North and Northeast regions, the predominance of respondents with more than 20 years of professional experience, and the absence of information regarding respondents’ primary dialysis modality may further limit the generalizability of the findings. Furthermore, the questionnaire underwent qualitative review and pilot testing but was not subjected to formal psychometric validation. Finally, the exploratory design and lack of stratified sampling precluded robust subgroup analyses by professional category, geographic region, and healthcare funding model. Despite these limitations, this study represents the first national survey to systematically assess sustainability-related practices and perceptions in Brazilian nephrology, providing valuable insights into current challenges and opportunities for advancing sustainable kidney care.

## CONCLUSION

The findings highlight important gaps between awareness, knowledge, and the implementation of sustainable practices in Brazilian nephrology, underscoring the need for structured strategies that integrate public policies, institutional incentives, and professional training.

The incorporation of sustainability into kidney care should be understood not only as an environmental imperative but also as a strategic opportunity to enhance efficiency, resilience, and equity within the healthcare system.

In this context, this study provides relevant evidence to support policy planning and interventions. Although the challenges are global, overcoming them requires approaches tailored to the context of the Brazilian Unified Health System and the country’s regional diversity.

## Data Availability

The data supporting the findings of this study are available from the corresponding authors upon reasonable request.

## SUPPLEMENTARY MATERIAL

The following online material is available for this article:

Supplementary Material – National Survey on Sustainable Nephrology – Brazil.
