# Supplementary material for: Knowledge and practices on sustainability in nephrology in Brazil
Source: J Bras Nefrol. 2026 Nov 9;49(1):e20260110. doi: 10.1590/2175-8239-JBN-2026-0110en (PMC13754508; doi:10.1590/2175-8239-JBN-2026-0110en)
Supplement: Supplementary file 1 [file 2175-8239-jbn-49-1-e20260110-en-suppl1.pdf]

Braz. J. Nephrol.
DOI: https://doi.org/10.1590/2175-8239-JBN-2026-0110en

Supplementary Material to "Knowledge and practices on sustainability in nephrology in Brazil"

National Survey on Sustainable Nephrology – Brazil

1. Number of participants: 180

2. Number of states represented:

20 states and the Federal District

3. Regional distribution

Region	N	%
North	9	5.0
Northeast	17	9.4
Midwest	13	7.2
Southeast	74	41.1
South	67	37.2
**Total**	180	100.0

4. Participant occupation

Position	N	%
Nephrologist	91	50.6
Nurse	29	16.1
Administrative assistant	21	11.7
Dialysis unit technical manager (nurse)	21	11.7
Nursing technician	7	3.9
Other (administrator, social worker, biomedical scientist, manager, non-nephrologist physician, nutritionist, etc.)	11	6.1
**Total**	180	100.0

5. How long have you been working in nephrology?

Years of experience	N	%
Less than 1 year	6	3.3
1–5 years	23	12.8
6–10 years	18	10.0
11–20 years	43	23.9
More than 20 years	90	50.0

6. What is the main funding source of the nephrology and dialysis center where you work?

Funding source	N	%
Only public system (SUS)	36	20.0
Only private/insurance	23	12.8
Mainly SUS	85	47.2
Mainly private/insurance	19	10.6
Philanthropic	16	8.9

7. Are you concerned about environmental impact and climate change?

Response	N	%
Not concerned	0	0
Slightly concerned	77	42.8
Neutral	6	3.3
Concerned	92	51.1
Very concerned	5	2.8

8. Do you believe that climate change may impact the health of patients with kidney disease?

Response	N	%
No impact	1	0.6
Little impact	8	4.4
Neutral	5	2.8
Some impact	63	35.0
Strong impact	103	57.2

9. What is your level of knowledge regarding the impact of climate change on kidney health?

Response	N	%
No knowledge	8	4.4
Little knowledge	61	33.9
Moderate knowledge	87	48.3
High knowledge	17	9.4
Expert-level knowledge	7	3.9

10. Are you aware of any public health policy related to climate change?

Response	N	%
Yes	64	35.6
No	115	63.9

11. In the center where you work, is there any sustainable measure/initiative?

Response	N	%
Yes	115	63.9
No	37	20.6
I do not have this information	27	15.0

12. In the center where you work, is there any type of awareness or education on sustainability and the environment?

Response	N	%
Yes	116	64.4
No	48	26.7
I do not have this information	15	8.3

13. Is environmental impact considered in decisions regarding resources, consumption, or transportation?

Response	N	%
Yes	102	56.7
No	50	27.8
I do not have this information	26	14.4

14. Regarding financial investment in measures to reduce environmental impact and optimize resource use, how do you assess the financial return?

Response	N	%
Investment with no return	9	5.0
Long-term savings	70	38.9
Medium-term savings	51	28.3
Short-term savings	12	6.7
I do not have this information	37	20.6

15. Select the measures adopted in your center regarding solid waste

Practice	N	%
Separation of recyclable waste	119	66.1
Recycling bins available	112	62.2
Proper waste disposal	142	78.9
Measures to reduce waste generation	96	53.3
Preference for recyclable packaging	50	27.8
Preference for biodegradable materials	42	23.3
Reduction and conscious use of paper	105	58.3
No measures adopted	6	3.3
I do not have this information	9	5.0

16. Select the water sources and practices adopted in your center

Practice	N	%
Use of well water	30	16.8
Municipal water supply	124	69.3
Rainwater harvesting	13	7.3
Reuse of rejected water	55	30.7
Additional osmotic treatment	32	17.9
Water-saving measures	72	40.2
Water-efficient faucets	60	33.5
No measures adopted	6	3.4
I do not have this information	14	7.8

17. Select the energy-related practices adopted in your center

Practice	N	%
Photovoltaic (solar) energy	53	29.6
Public electricity utility	113	63.1
Renewable energy provider	19	10.6
Energy-efficient lighting	101	56.4
Turning off unused devices	98	57.4
Motion sensors	50	27.9
Energy-efficient equipment	58	32.4
Other energy-saving measures	26	14.5
Low-impact transportation options	5	2.8
Thermal comfort/climate control	81	45.3
No measures adopted	6	3.4
I do not have this information	11	6.1

18. Select the three main barriers to implementing sustainability measures

Barrier	N	%
Financial resources	122	68.5
Lack of human resources	24	13.5
Limited knowledge	57	32.0
Lack of adequate technology	35	19.7
Lack of strategic planning	70	39.3
Lack of government incentives	110	61.8
Lack of local support	49	27.5
Other	13	7.3

19. Describe sustainable actions implemented in your center (open-ended responses)

Waste:

- Reuse of dialyzers and lines
- Recycling of containers
- Sale/reuse of HDPE containers
- Recycling programs
- Use of biodegradable materials
- Reduction of contaminated waste

Water:

- Reuse of reverse osmosis reject water
- Rainwater harvesting
- Water monitoring and management

Energy:

- Renewable energy use
- Equipment modernization
- Energy efficiency measures

General measures:

- Environmental education programs
- Paperless systems
- Process optimization
- Staff training

20. Do you adopt personal measures to reduce environmental impact?

Response	N	%
Yes	131	89.4
No	16	8.9

21. Have you heard of "Green Nephrology"?

Response	N	%
Yes	80	44.4
No	98	54.4

22. Do you think more information on sustainable nephrology is needed?

Response	N	%
Yes	176	97.8
No	2	1.1
