# Supplementary material for: Knowledge and practices on sustainability in nephrology in Brazil
Source: J Bras Nefrol. 2026 Nov 9;49(1):e20260110. doi: 10.1590/2175-8239-JBN-2026-0110en (PMC13754508; doi:10.1590/2175-8239-JBN-2026-0110en)
Supplement: Supplementary file 2 [file 2175-8239-jbn-49-1-e20260110-pt-suppl1.pdf]

Braz. J. Nephrol.
DOI: https://doi.org/10.1590/2175-8239-JBN-2026-0110pt

Material Suplementar para "Conhecimento e práticas sobre sustentabilidade em nefrologia no Brasil"

Questionário sobre Conhecimento e Práticas sobre Sustentabilidade em Nefrologia no Brasil

1. Número de participantes: 180

2. Número de estados dos participantes: 20 e DF

3. Distribuição por região:

Região	N	Percentual (%)
Norte	9	5,0
Nordeste	17	9,4
Centro-Oeste	13	7,2
Sudeste	74	41,1
Sul	67	37,2
**Total**	180	100,0

4. Ocupação dos participantes

Cargo	N	%
Médico(a) Nefrologista	91	50,6
Enfermeiro(a)	29	16,1
Assistente administrativo	21	11,7
Responsável técnico de unidade de diálise (enfermeiros)	21	11,7
Técnico(a) de Enfermagem	7	3,9
Outros (Administrador(a), Assistente social, Biomédica, Gerente, Médico não nefrologista, Nutricionista, etc.)	11	6,1
**Total**	180	100,0

5. Há quanto tempo atua em Nefrologia?

**Tempo de atuação**	**N**	**%**
Menos de 1 ano	6	3,3
Entre 1 e 5 anos	23	12,8
Entre 6 e 10 anos	18	10,0
Entre 11 e 20 anos	43	23,9
Mais de 20 anos	90	50,0

6. Qual a principal financiadora do centro de Nefrologia e diálise em que atua?

	**N**	**%**
Apenas SUS	36	20
Apenas particular/convênios	23	12,8
Principalmente SUS	85	47,2
Principalmente particular/convênios	19	10,6
Filantrópico	16	8,9

7. Você se preocupa com impacto ambiental e mudanças climáticas?

	**N**	**%**
Não me preocupo	0	0
Me preocupo pouco	77	42,8
Sou neutro	6	3,3
Me preocupo	92	51,1
Me preocupo muito	5	2,8

8. Você acredita que as mudanças climáticas possam ter impacto na saúde dos pacientes com doença renal?

	**N**	**%**
Nenhum impacto	1	0,6
Pouco impacto	8	4,4
Sou neutro	5	2,8
Algum impacto	63	35
Muito impacto	103	57,2

9. Qual seu nível de conhecimento em relação ao impacto das mudanças climáticas na saúde dos pacientes com doença renal?

	**N**	**%**
Não tenho conhecimento	8	4,4
Tenho pouco conhecimento	61	33,9
Tenho conhecimento	87	48,3
Tenho muito conhecimento	17	9,4
Tenho domínio sobre o tema	7	3,9

10. Você conhece alguma política pública na área da saúde sobre mudança climática?

	**N**	**%**
Sim	64	35,6
Não	115	63,9

11. **No centro em que você atua existe alguma medida/iniciativa sustentável?**

	**N**	**%**
Sim	115	63,9
Não	37	20,6
Não tenho essa informação	27	15,0

12. Na clínica em que atua existe algum tipo de conscientização ou educação sobre sustentabilidade e meio ambiente?

	N	%
Sim	116	64,4
Não	48	26,7
Não tenho essa informação	15	8,3

13. O impacto ambiental é levado em consideração nas decisões referente a recursos, consumo ou transporte.

	N	%
Sim	102	56,7
Não	50	27,8
Não tenho essa informação	26	14,4

14. Com relação a investimento financeiro em medidas para reduzir impacto ambiental e melhor uso de recursos. De uma forma geral, como você avalia o retorno financeiro deste investimento?

	N	%
Investimento sem retorno	9	5,0
Investimento com economia a longo prazo	70	38,9
Investimento com economia a médio prazo	51	28,3
Investimento com economia a curto prazo	12	6,7
Não tenho essa informação	37	20,6

15. Selecione as medidas adotadas pelo centro em que atua com relação a resíduos sólidos

	N	%
Realiza separação do lixo reciclável	119	66,1
Possui lixeiras de coleta seletiva (plástico, vidro, papel, orgânico)	112	62,2
Realiza encaminhamento adequado de resíduos.	142	78,9
Realiza medidas para redução do consumo de resíduos.	96	53,3
Prioriza consumo de produtos com embalagens recicláveis.	50	27,8
Preferência pelo uso de material biodegradável, quando essa opção está disponível.	42	23,3
Redução e uso consciente de papel.	105	58,3
Não adotamos nenhuma medida.	6	3,3
Não tenho essa informação.	9	5

16. Selecione a fonte de recursos utilizados e as medidas adotadas, no centro de nefrologia em que atua, referentes ao uso de água.

	N	%
Utiliza água de poço.	30	16,8
Utiliza companhia de abastecimento de água.	124	69,3
Realiza captação de água pluvial.	13	7,3
Utiliza água descartada de rejeito no tratamento de água para outra finalidade.	55	30,7
Utiliza água descartada de rejeito no tratamento de água com osmose adicional.	32	17,9
Realiza medidas para economia de água.	72	40,2
Possui torneira com válvulas para economia de água.	60	33,5
Não adotamos nenhuma medida.	6	3,4
Não tenho essa informação.	14	7,8

17. Selecione as medidas adotadas pelo centro em que atua com relação ao uso de energia e combustíveis.

	N	%
Utiliza energia fotovoltaica (solar).	53	29,6
Utiliza concessionária de energia elétrica.	113	63,1
Utiliza provedor de energia renovável.	19	10,6
Utiliza lâmpadas de baixo consumo de energia.	101	56,4
Prioriza desligar aparelhos eletrônicos quando não estão em uso.	98	57,4
Utiliza sensor de presença em luzes.	50	27,9
Utiliza aparelhos com melhor eficiência energética. Outras medidas para economizar energia.	58	32,4
Outras medidas para economizer energia	26	14,5
Opções (alternativas) de transporte para pacientes e colaboradores com redução de impacto ambiental.	5	2,8
Preocupações com climatização do ambiente, conforto térmico.	81	45,3
Não adotamos nenhuma medida.	6	3,4
Não tenho essa informação.	11	6,1

18. Selecione as três principais barreiras e sua influência na implementação de medidas para reduzir o impacto ambiental no centro de Nefrologia e/ou diálise.

	N	%
Recurso financeiro.	122	68,5
Falta de recursos humanos.	24	13,5
Pouco conhecimento sobre o assunto.	57	32
Ausência de tecnologia adequada.	35	19,7
Ausência de plano estratégico.	70	39,3
Ausência de incentivo pelo ministério da saúde e órgãos federados.	110	61,8
Ausência de apoio local.	49	27,5
Outras:	13	7,3

19. Descreva ou exemplifique ações e implementação de medida sustentável no centro de Nefrologia e/ou diálise que atua.

Ações novas encontradas nas respostas abertas

(ou seja, não aparecem explicitamente nas opções de múltipla escolha)

Resíduos:

- Reuso de capilares e linhas.
- Reciclagem de galões de banho.
- Venda/reaproveitamento de bombonas de concentrado de hemodiálise (CPHD).
- Encaminhamento de tampas de galões para reciclagem solidária.
- Incineração de descartáveis para geração de energia.
- Uso de canecas/térmicas individuais para evitar copos descartáveis.
- Uso de materiais biodegradáveis nos lanches dos pacientes.
- Segregação rigorosa de resíduos biológicos para não contaminar recicláveis.
- Farmácia própria que evita descarte excessivo de bombonas plásticas.
- Drenar capilar ao término da terapia para reduzir volume de lixo contaminado.

Água:

- Reutilização do rejeito da osmose em banheiros, lavanderia, limpeza e outros setores.
- Reaproveitamento da água da chuva (alguns já citaram, mas aqui aparecem usos específicos).
- Monitoramento da qualidade da água do poço (além do simples uso).
- Sistema automatizado de tratamento de água e reuso.
- Gestão racional do uso da água.
- Redução do consumo de banhos de diálise (equipamentos com adapt flow).

Energia:

- Contrato de energia no Mercado Livre (compra de energia limpa mais barata).
- Compra de energia eólica.
- Modernização do parque de máquinas e equipamentos para eficiência.
- Manutenção de ar-condicionado em dia.
- Uso de paredes/pisos para melhorar isolamento térmico (ambiente mais eficiente).
- Projeto de pesquisa sobre impacto das mudanças climáticas.

Medidas gerais/transversais (educação e gestão):

- Campanhas internas/educação ambiental para colaboradores e pacientes.
- Conscientização sobre consumo de insumos.
- Racionalização de processos de trabalho para reduzir consumo e resíduos.
- Uso de prontuário eletrônico/tablets ("hospital sem papel").
- Redução do uso de papel com reaproveitamento de folhas para rascunho.
- Zero papel nos salões de diálise.
- Dedetização e limpeza regular das caixas de água (manutenção preventiva).
- Treinamentos sobre descarte adequado.

20. Você adota em sua rotina pessoal medidas para minimizar o impacto ambiental?

	**N**	**%**
Sim	131	89,4
Não	16	8,9

21. Você já ouviu falar dos termos "Green Nephrology" e "Nefrologia Verde"?

	**N**	**%**
Sim	80	44,4
Não	98	54,4

22. Você acha que falta mais informações sobre nefrologia sustentável e medidas para otimizar recursos?

	**N**	**%**
Sim	176	97,8
Não	2	1,1
