# Supplementary material for: Knowledge and practices on sustainability in nephrology in Brazil
Source: J Bras Nefrol. 2026 Nov 9;49(1):e20260110. doi: 10.1590/2175-8239-JBN-2026-0110en (PMC13754508; doi:10.1590/2175-8239-JBN-2026-0110en)
Supplement: Translation

# Translation: Conhecimento e práticas sobre sustentabilidade em nefrologia no Brasil

**DOI:** 10.1590/2175-8239-JBN-2026-0110pt

**Authors:** Fabiana Baggio Nerbass, Nandressa D. Mendes Riso, Cinthia Kruger Sobral Vieira, André L Pimentel, Ana Flavia Moura, Luis Claudio Pinto, Fátima R. Veiga Tostes, Rafael B. Trancoso, Talita G. Salani, José A. Moura-Neto

**Affiliations:** 1 Sociedade Brasileira de Nefrologia, São Paulo, SP, Brasil.; 2 Fundação Pró-Rim, Joinville, SC, Brasil.; 3 Hospital Universitário do Oeste do Paraná, Cascavel, PR, Brasil.; 4 Clinefro Serviço de Nefrologia e Diálise, Porto Alegre, RS, Brasil.; 5 Associação Brasileira de Centros de Diálise Transplantes, Brasília, DF, Brasil.; 6 Escola Bahiana de Medicina e Saúde Pública, Salvador, BA, Brasil.; 7 Universidade Federal do Pará, Belém, PA, Brasil.; 8 Hospital São Vicente de Paulo, Rio de Janeiro, RJ, Brasil..; 9 Hospital Santa Rita, Vitória, ES, Brasil.

**Keywords:** Nefropatias, Diálise Renal, Mudança Climática, Meio Ambiente

## Abstract

**Introdução:**

As mudanças climáticas apresentam uma relação bidirecional com as doenças renais: enquanto fatores ambientais podem aumentar a incidência e a progressão dessas doenças, a terapia dialítica está entre as modalidades de tratamento com maior impacto ambiental na área da saúde. Apesar do crescente debate internacional sobre nefrologia sustentável, esse tema permanece pouco explorado no contexto brasileiro.

**Objetivo:**

Investigar o grau de conhecimento, as percepções e as práticas relacionadas à sustentabilidade ambiental entre profissionais atuantes na nefrologia no Brasil, bem como identificar barreiras à adoção de estratégias sustentáveis nos serviços de diálise.

**Métodos:**

Estudo transversal realizado por meio de questionário online direcionado a profissionais da nefrologia. O instrumento abordou conhecimentos sobre mudanças climáticas e saúde renal, práticas sustentáveis adotadas nos serviços e barreiras institucionais à sua implementação.

**Resultados:**

Foram analisadas 180 respostas de profissionais de 20 estados brasileiros e do Distrito Federal, com predomínio de nefrologistas e enfermeiros. A maioria reconheceu a influência das mudanças climáticas sobre a saúde renal; contudo, observou-se conhecimento limitado sobre políticas públicas e estratégias estruturadas de sustentabilidade. As práticas mais frequentemente relatadas envolveram o manejo de resíduos, enquanto iniciativas como reúso de água, uso de fontes de energia renovável e estratégias de transporte sustentável foram menos prevalentes. As principais barreiras identificadas incluíram restrições financeiras, ausência de incentivos institucionais e falta de planejamento estratégico.

**Conclusão:**

Os achados evidenciam lacunas entre percepção, conhecimento e implementação de práticas sustentáveis na nefrologia brasileira, destacando a necessidade de políticas, capacitação profissional e estratégias institucionais voltadas à sustentabilidade ambiental no cuidado nefrológico.

## INTRODUÇÃO

As mudanças climáticas configuram uma ameaça crescente à saúde e ao bem-estar da humanidade. Segundo a Organização Mundial da Saúde, as variações no clima já estão associadas a emergências humanitárias provocadas por ondas de calor, incêndios florestais, inundações, tempestades tropicais e furacões, cuja frequência e intensidade vêm aumentando nas últimas décadas^1^.

As doenças renais e as mudanças climáticas mantêm uma relação bidirecional, em que cada uma pode potencializar os efeitos da outra^2^. O aumento das temperaturas está associado à maior incidência de litíase, injúria renal aguda e à progressão da doença renal crônica^3^. Por outro lado, o tratamento da doença renal, particularmente por meio da diálise, apresenta uma das maiores pegadas ambientais entre as modalidades de cuidado em saúde, em virtude do elevado consumo de energia, água e materiais plásticos descartáveis^4^.

Nos últimos anos, a nefrologia tem sido progressivamente reconhecida como uma área estratégica na agenda global de sustentabilidade em saúde. Considerando o cenário atual, espera-se um aumento importante na quantidade de pessoas em tratamento dialítico e, portanto, no consumo de água e energia, bem como na geração de carbono pelo setor no Brasil^5,6^. Análises internacionais comparativas demonstram que o impacto ambiental e a organização do cuidado renal variam amplamente entre países, refletindo diferenças estruturais, econômicas e regulatórias nos sistemas de saúde^7^. Nesse contexto, têm surgido posicionamentos e iniciativas em âmbito global, enquanto diversas sociedades de nefrologia têm publicado artigos e recomendações sobre nefrologia e diálise sustentáveis, refletindo o crescente reconhecimento do impacto ambiental dessa terapia renal^8,9,10^. A Sociedade Internacional de Nefrologia lançou a iniciativa “GREEN-K”(*Global Environmental Evolution in Nephrology and Kidney Care*), cuja proposta enfatiza a necessidade de fortalecer estratégias de promoção da saúde, prevenir e retardar a progressão da doença renal, ampliar o acesso ao transplante e desenvolver terapias dialíticas ambientalmente mais responsáveis. O documento reforça, ainda, que a educação e o engajamento dos profissionais são fundamentais para a implementação de práticas sustentáveis^2^.

No Brasil, esse movimento tem avançado nos últimos anos^11^. Em 2023, a Sociedade Brasileira de Nefrologia (SBN) criou o Comitê de Nefrologia Sustentável, responsável pela elaboração de recomendações nacionais voltadas à incorporação de práticas sustentáveis no cuidado renal. Em 2025, essas recomendações foram apresentadas e aprovadas na 2ª Convenção da Diretoria Plena da SBN e suas Regionais. O documento consolida dez recomendações estratégicas, com implementação progressiva prevista para o período de 2027 a 2030, representando um marco institucional na agenda de sustentabilidade da nefrologia brasileira^12^. Adicionalmente, a temática da sustentabilidade tem sido progressivamente incorporada à agenda científica danefrologia brasileira. Os Congressos Brasileiros de Nefrologia de 2022 e 2024 incluíram o tema em sua programação científica. A edição de 2024, realizada em Salvador (BA), representou um marco ao adotar a mensuração da pegada de carbono do evento, com compensação de 128 toneladas de CO_2_ equivalente (tCO_2_e) por meio do Projeto de Manutenção Florestal Envira Amazônia^13^.

Apesar desses avanços, o conhecimento dos profissionais de saúde sobre sustentabilidade em nefrologia ainda é limitado. Uma pesquisa que incluiu cerca de mil profissionais de 108 países revelou que menos de 50% possuíam conhecimento sobre o efeito das mudanças climáticas na saúde renal ou sobre o impacto ambiental dos cuidados renais, e apenas 26% relataram trabalhar em organizações com iniciativas sustentáveis^14^. Inquéritos realizados em outros países demonstram que essas lacunas de conhecimento e a implementação heterogênea de práticas sustentáveis em nefrologia não se restringem a países de baixa ou média renda, sendo observadas também em sistemas de saúde consolidados, como os do Canadá, de países europeus e do Japão^15,16,17^. Não existem dados que avaliem essas questões junto à comunidade nefrológica no Brasil.

Assim, este estudo visou mapear o conhecimento, as atitudes e as práticas dos profissionais de saúde com atuação na nefrologia em relação à interconexão entre as mudanças climáticas e a saúde renal, além de identificar iniciativas e práticas organizacionais sustentáveis.

## MÉTODO

### Desenho do estudo

Este foi um estudo transversal, conduzido por meio de um questionário online, desenvolvido por membros do Comitê de Nefrologia Sustentável e da diretoria da SBN. O instrumento foi revisado por outros membros do Comitê de Nefrologia Sustentável com experiência em sustentabilidade em nefrologia e metodologia de pesquisa. Foi realizado um estudo piloto com profissionais de saúde de diferentes áreas de atuação para avaliar a clareza, a compreensão e a adequação do conteúdo. Esse processo constituiu uma avaliação qualitativa do instrumento, não tendo sido realizada validação psicométrica formal nem avaliação quantitativa da validade de conteúdo ou de construto.

O questionário foi divulgado por múltiplos canais, incluindo convites enviados por e-mail aos membros da SBN e aos endereços eletrônicos cadastrados pelas unidades de diálise, além das plataformas institucionais de redes sociais e grupos em aplicativos de mensagens. Não foram estabelecidas cotas nem amostragem estratificada por categoria profissional. A participação foi voluntária e aberta a todos os profissionais de saúde envolvidos no cuidado em nefrologia. As respostas foram coletadas entre maio e julho de 2025, e o questionário completo está apresentado no material suplementar.

### População-alvo

A pesquisa foi direcionada a profissionais de saúde envolvidos no cuidado de pacientes com doença renal, incluindo médicos nefrologistas, enfermeiros, técnicos e outros profissionais da área. Não foram estabelecidos critérios de exclusão relacionados ao local de trabalho ou ao tempo de experiência profissional.

### Coleta de dados

Os participantes responderam ao questionário online por meio da ferramenta Google Forms. O instrumento incluiu questões estruturadas que abordaram: (1) características sociodemográficas e profissionais; (2) conhecimento sobre mudanças climáticas e sua relação com a saúde renal; (3) práticas sustentáveis adotadas nos serviços; e (4) barreiras institucionais à implementação de estratégias sustentáveis (material suplementar).

### Considerações éticas

O estudo foi conduzido em conformidade com os princípios éticos que regem pesquisas envolvendo seres humanos. Não foram solicitadas informações que permitissem a identificação pessoal ou do local de trabalho. O estudo foi aprovado pelo Comitê de Ética em Pesquisa do Hospital Ernesto Dornelles (parecer nº 7.461.891, de 25 de março de 2025), sob o Certificado de Apresentação para Apreciação Ética (CAAE) nº 86807325.2.0000.5304.

## RESULTADOS

### Características dos participantes

Foram incluídos no estudo 180 respondentes. Houve participação de profissionais de 20 estados brasileiros e do Distrito Federal, com predominância da região Sudeste (41%), seguida pelas regiões Sul (37%), Nordeste (9%), Centro-Oeste (7%) e Norte (5%).

Entre os participantes, predominaram médicos nefrologistas (51%), seguidos por enfermeiros (28%) e outros profissionais da área (21%). Metade dos respondentes possuía mais de 20 anos de atuação em nefrologia, enquanto 24% tinham entre 11 e 20 anos de experiência e 26%, menos de 10 anos.

Quanto à fonte de financiamento da unidade de diálise em que atuavam, 67% relataram que o atendimento era financiado exclusivamente ou predominantemente pelo Sistema Único de Saúde (SUS).

### Percepções sobre mudanças climáticas e saúde renal

Pouco mais da metade dos respondentes (54%) relatou ter um maior nível de preocupação com o impacto ambiental e as mudanças climáticas. Os que se preocupam pouco ou são neutros somaram 46%. Quanto à relação entre mudanças climáticas e saúde de pacientes com doença renal, 57% acreditam haver muito impacto, 35%, algum impacto, e os que se declararam neutros ou que percebem pouco ou nenhum impacto somaram 8%. Em relação ao nível de conhecimento sobre o tema, 38% declararam ter pouco ou nenhum conhecimento, 48% responderam ter algum conhecimento e 13%, ter muito conhecimento. Apenas 36% afirmaram conhecer alguma política pública na área da saúde voltada às mudanças climáticas.

### Práticas de sustentabilidade nas unidades de diálise

A Tabela 1 apresenta três questões relacionadas à sustentabilidade nos centros e clínicas de atuação. De forma geral, a maioria dos respondentes indica a existência de iniciativas sustentáveis, programas de conscientização ou a consideração do impacto ambiental nas decisões, embora uma proporção significativa ainda não possua essa informação ou não adote tais práticas.

**Tabela 1:** Práticas e percepção de sustentabilidade em clínicas de diálise.

Pergunta em relação à unidade de diálise	Sim	Não	Não sei
Existe alguma medida/iniciativa sustentável?	64%	21%	15%
Existe algum tipo de conscientização ou educação sobre sustentabilidade e meio ambiente?	64%	27%	8%
O impacto ambiental é levado em consideração nas decisões referentes a recursos, consumo ou transporte?	57%	28%	14%

As principais práticas em relação a resíduos, água e energia utilizadas pelos centros de diálise estão descritas na Tabela 2. Entre as relacionadas a resíduos, as mais frequentes foram o encaminhamento adequado de resíduos (79%), a separação de resíduo reciclável (66%) e a disponibilidade de lixeiras de coleta seletiva (62%). Quanto ao uso de água, a maioria utiliza água de companhia de abastecimento (69%) e adota medidas de economia de água (40%), incluindo torneiras com válvulas econômicas (34%) e reaproveitamento de água descartada (31%). Em relação à energia e à iluminação, as medidas mais adotadas incluem desligar aparelhos quando não utilizados (57%) e utilizar lâmpadas de baixo consumo (56%). Medidas de menor adesão incluem energia solar, provedores de energia renovável e transporte com menor impacto ambiental.

**Tabela 2:** Práticas utilizadas pelos centros de diálise.

Recurso	Práticas	%
Resíduos	Realiza encaminhamento adequado de resíduos	79
	Realiza separação do lixo reciclável	66
	Possui lixeiras de coleta seletiva (plástico, vidro, papel, lixo orgânico)	62
	Redução e uso consciente de papel	58
	Adota medidas para reduzir a geração de resíduos	53
	Prioriza o consumo de produtos com embalagens recicláveis	28
	Preferência pelo uso de material biodegradável, quando disponível	23
	Não adotamos nenhuma medida	3
	Não tenho essa informação	5
Água	Utiliza água de companhia de abastecimento	69
	Adota medidas para economia de água	40
	Possui torneiras com válvulas para economia de água	34
	Utiliza água de rejeito descartada no tratamento de água para outra finalidade	31
	Utiliza água de rejeito descartada no tratamento de água, com osmose adicional	18
	Utiliza água de poço	17
	Realiza captação de água pluvial	7
	Não adotamos nenhuma medida	3
	Não tenho essa informação	8
Energia	Utiliza energia elétrica de concessionária	63
	Prioriza desligar aparelhos eletrônicos quando não estão em uso	57
	Utiliza lâmpadas de baixo consumo de energia	56
	Preocupação com a climatização do ambiente e o conforto térmico	45
	Utiliza aparelhos com melhor eficiência energética ou adota outras medidas para economizar energia	32
	Utiliza energia fotovoltaica (solar)	30
	Utiliza sensores de presença para iluminação	28
	Outras medidas para economizar energia	15
	Utiliza energia proveniente de fornecedor de fontes renováveis	11
	Opções de transporte com redução do impacto ambiental	3
	Não adotamos nenhuma medida	3
	Não tenho essa informação	6

Nota – Os percentuais podem não totalizar 100%, pois os respondentes puderam selecionar mais de uma opção.

Outras ações descritas pelos participantes estão no material suplementar.

### Sustentabilidade, economia e barreiras à implementação

Sobre a percepção do retorno financeiro de investimentos em sustentabilidade, a maioria dos participantes indicou quemedidas para reduzir o impacto ambiental e otimizar o uso de recursos trazem economia, principalmente no longo prazo (39%) e no médio prazo (28%). Uma parcela menor acredita que o retorno ocorre no curto prazo (7%) ou que o investimento não gera retorno (5%). Cerca de 21% dos respondentes não tinham informações sobre o tema.

Os participantes identificaram a falta de recursos financeiros (68%) e a ausência de incentivo do Ministério da Saúde e de órgãos federados (62%) como as principais barreiras para a implementação de medidas que reduzam o impacto ambiental. Outras dificuldades relevantes incluíram a ausência de plano estratégico (39%), pouco conhecimento sobre o tema (32%) e ausência de apoio local (27%). Barreiras secundárias foram a falta de recursos humanos (13%), a ausência de tecnologia adequada (20%) e outras mencionadas por 7% dos respondentes.

## DISCUSSÃO

Os resultados deste estudo revelam que, embora haja uma crescente percepção sobre a relação entre mudanças climáticas, sustentabilidade e saúde renal, persistem lacunas importantes no conhecimento, na implementação de práticas e no apoio institucional nas unidades de diálise brasileiras.

Resultados semelhantes foram observados em um levantamento realizado no Canadá, no qual profissionais de nefrologia também demonstraram lacunas importantes de conhecimento sobre estratégias de cuidado renal ambientalmente sustentável, especialmente em áreas relacionadas a políticas institucionais, uso de energia e gestão hídrica, mesmo em um contexto de sistema de saúde melhor estruturado^18^.

Nossa amostra incluiu profissionais de diversas regiões do país, com predomínio das regiões Sudeste e Sul. A maior parte dos respondentes era formada por médicos nefrologistas, com ampla experiência na área. Além disso, a maioria atuava em unidades financiadas majoritariamente pelo SUS, cenário que pode influenciar diretamente as possibilidades de investimento e implementação de práticas sustentáveis.

Metade dos respondentes possuía mais de 20 anos de experiência em nefrologia. Essa característica pode ter influenciado os achados, uma vez que profissionais em diferentes estágios da carreira podem diferir quanto à familiaridade com os conceitos de sustentabilidade, às responsabilidades institucionais e à percepção dos desafios ambientais. Embora profissionais mais experientes possam oferecer contribuições valiosas sobre as mudanças de longo prazo no cuidado em nefrologia, profissionais mais jovens podem ter sido expostos com maior frequência a temas relacionados à sustentabilidade durante sua formação acadêmica e treinamento profissional.

### Percepções sobre mudanças climáticas e saúde renal

Os resultados indicam que os profissionais de nefrologia reconhecem o impacto das mudanças climáticas sobre a saúde de pacientes com doença renal, embora esse reconhecimento ainda não se traduza em conhecimento aprofundado sobre o tema. De fato, a literatura tem evidenciado que, apesar do crescimento acelerado da produção científica sobre o impacto do clima na nefrologia, a integração desse conhecimento à prática clínica e ao planejamento de políticas de saúde ainda é limitada. A maioria dos profissionais reconhece o problema, mas poucos têm formação específica ou acesso a protocolos adaptados para mitigação e adaptação dos cuidados nefrológicos diante das mudanças climáticas^2,19^.

Dois achados adicionais merecem atenção especial. Primeiro, quase todos os respondentes (97,8%) indicaram haver necessidade de mais informações sobre nefrologia sustentável e estratégias para otimização de recursos. Esse resultado evidencia uma importante demanda não atendida por educação e capacitação nessa área entre os profissionais de nefrologia. Em segundo lugar, menos da metade dos participantes (44,4%) relatou estar familiarizada com os termos “Nefrologia Verde” ou “Nefrologia Sustentável”, apesar da criação do Comitê de Nefrologia Sustentável da SBN em 2023. Em conjunto, esses achados sugerem que, embora a sustentabilidade tenha ganhado visibilidade nas agendas científica e institucional, os esforços de conscientização e disseminação do tema ainda não alcançaram uma parcela expressiva da comunidade nefrológica brasileira.

### Práticas de sustentabilidade nas unidades de diálise

A maior parte dos participantes relatou a presença de iniciativas sustentáveis, ações educativas e a consideração do impacto ambiental nas decisões das unidades, embora ainda exista uma parcela relevante que desconhece ou não adota tais práticas. Essa heterogeneidade é consistente com estudos qualitativos e quantitativos que demonstram que o engajamento em práticas sustentáveis varia amplamente entre diferentes contextos e perfis profissionais, influenciado por fatores como nível de conhecimento, valores individuais, barreiras práticas e características organizacionais^20,21^. Especificamente na área da nefrologia, organizações internacionais que propõem ações globais para promover práticas sustentáveis, educação e colaboração em nefrologia reconhecem que a implementação é heterogênea e que muitos profissionais ainda não estão engajados ou informados sobre sustentabilidade^2,8,16^.

### Manejo de resíduos

Os achados deste estudo demonstram que as práticas relacionadas ao manejo de resíduos aparecem como algumas das ações sustentáveis mais consolidadas nas unidades de diálise. A elevada frequência de relatos sobre a separação e o encaminhamento adequados de resíduos recicláveis indica que os centros já incorporam procedimentos básicos de gestão de resíduos, um aspecto relevante dada a grande quantidade de material descartável utilizada na diálise. Iniciativas de uso consciente de papel reforçam essa tendência, embora práticasmais avançadas, como a priorização de embalagens recicláveis ou o uso de materiais biodegradáveis, ainda permaneçam pouco frequentes. Essa heterogeneidade é consistente com o cenário nacional, no qual a taxa geral de reciclagem ainda é baixa (8,3% em 2023)^22^ e a implementação depende de infraestrutura local, logística e incentivos regulatórios. Uma iniciativa relatada por unidades de diálise no Brasil é a venda de bombonas de polietileno de alta densidade (PEAD) e das caixas de papelão utilizadas no acondicionamento desses insumos, destinando a receita obtida à compra de cestas básicas para pacientes em situação de vulnerabilidade social^23^.

### Manejo da água

Em relação ao uso de água, os resultados indicam que, embora o fornecimento seja amplamente assegurado nas unidades, a adoção de estratégias voltadas à economia e ao uso mais eficiente ainda é limitada do ponto de vista dos profissionais. O predomínio da utilização de água proveniente de companhias de abastecimento (69%) reflete a dependência de um suprimento contínuo e seguro, fundamental para o funcionamento dos sistemas de tratamento de água utilizados na diálise. Medidas de economia, contudo, foram relatadas por apenas 40% dos respondentes, e o relato, por cerca de um terço dos participantes, de práticas específicas, como o uso de torneiras com válvulas econômicas ou o reaproveitamento da água descartada no tratamento, sugere que há amplo espaço para expansão de iniciativas sustentáveis no manejo hídrico.

No Brasil, embora a RDC nº 11/2014 estabeleça normas para os serviços de diálise e para a qualidade da água utilizada na terapia renal substitutiva, a regulamentação nacional é limitada em relação ao reúso da água de rejeito da osmose reversa para fins não potáveis. Consequentemente, a implementação de estratégias de reúso de água permanece dependente da infraestrutura local, das políticas institucionais e dos protocolos operacionais, representando um desafio adicional para a adoção mais ampla de práticas sustentáveis de gestão da água nas unidades de diálise.

Cada sessão de hemodiálise consome, em média, cerca de 500 litros de água, e aproximadamente dois terços desse volume são rejeitados pelo sistema de osmose reversa e descartados na rede de esgoto^8^. Nesse contexto, estratégias de gestão circular, incluindo redução, reúso e reciclagem da água, têm sido recomendadas em publicações internacionais, embora sua implementação permaneça limitada globalmente^24,25^.

### Manejo de energia

No que diz respeito ao uso de energia, os achados evidenciam que medidas básicas de eficiência energética são mais frequentes do que estratégias estruturais de transição para fontes renováveis. A maioria dos respondentes relatou práticas simples, como desligar aparelhos quando não estão em uso e utilizar lâmpadas de baixo consumo, indicando que ações de fácil implementação já fazem parte da rotina de muitas unidades.

Por outro lado, práticas que exigem maior investimento financeiro ou alterações na infraestrutura permanecem menos relatadas: 30% mencionaram o uso de energia fotovoltaica, 11%, o acesso a provedores de energia renovável e 28% a utilização de sensores de presença. Estudos internacionais descrevem padrão semelhante. Em levantamento realizado na Austrália e na Nova Zelândia, cerca de 44% das unidades de diálise utilizavam sensores de movimento para iluminação, enquanto somente 14% relatavam o uso de energia renovável, seja por meio de painéis solares ou contratos verdes^25^. Diversas barreiras têm sido apontadas para explicar essa baixa adoção, incluindo restrições financeiras, falta de incentivos institucionais, ausência de políticas públicas específicas, limitações da infraestrutura física, desconhecimento técnico e baixa priorização das questões ambientais nas decisões administrativas^9,25^. Assim, há espaço significativo para ampliar o uso de estratégias estruturais e fontes renováveis nas unidades de diálise. Superar barreiras institucionais, econômicas e educacionais será fundamental para avançar na sustentabilidade energética e reduzir a pegada ambiental do cuidado renal.

Uma revisão de escopo recente que avaliou intervenções sustentáveis em cuidados renais reforça que, apesar do crescente interesse global no tema, a adoção de práticas sustentáveis permanece fragmentada, concentrada principalmente na hemodiálise e limitada pela ausência de métricas padronizadas, governança institucional e integração às políticas de saúde^26^.

### Sustentabilidade, economia e barreiras à implementação

A percepção dos participantes sobre a relação entre sustentabilidade e economia evidencia que grande parte das unidades reconhece o potencial de retorno financeiro dessas iniciativas, sobretudo quando consideradas em horizontes temporais mais amplos. Para a maior parte dos participantes, os benefícios econômicos tornam-se mais evidentes a médio ou a longo prazo, e cerca de um quinto declarou não ter informações suficientes para opinar. O custo financeiro foi citado como o principal entrave (68%), seguido pela ausência de incentivos institucionais por parte do Ministério da Saúde e de órgãos federados (62%). Adicionalmente, fatores organizacionais internos constituem obstáculos relevantes. A ausência de um plano estratégico para sustentabilidade (39%), a falta de conhecimento técnico sobre o tema (32%) e a percepção de pouco apoio local (27%) sugerem que processos de capacitação, sensibilização e governança podem desempenhar papel crucial para fortalecer a agenda ambiental nas unidades. Experiências internacionais mostram que a implementação de indicadores de desempenho ambiental, o apoio financeiro para atualização tecnológica e as políticas públicas voltadas à sustentabilidade são determinantes para ampliar o impacto e a adesão às práticas sustentáveis^9,27^.

Estudos europeus apontam que a existência de estruturas organizacionais formais, como equipes dedicadas à sustentabilidade, denominadas “Green Teams”, está associada a uma maior implementação de práticas ambientais em unidades de diálise, sugerindo que o engajamento institucional é determinante para transformar conhecimento e preocupação ambiental em ações concretas^16^.

### Pontos fortes e limitações

As principais limitações deste estudo incluem o uso de amostragem por conveniência e a participação voluntária, o que introduz potencial para vieses de seleção, de não resposta e de voluntariado. Consequentemente, profissionais com maior interesse em sustentabilidade ambiental podem ter sido mais propensos a participar, potencialmente superestimando o nível de conhecimento, engajamento e adoção de práticas sustentáveis. Além disso, como não foram estabelecidas cotas nem foi utilizada amostragem estratificada por categoria profissional, a distribuição observada entre os grupos profissionais (51% nefrologistas, 28% enfermeiros e 21% outros profissionais) provavelmente reflete diferenças no acesso e no engajamento com os canais de divulgação utilizados, e não a composição real da força de trabalho em nefrologia no Brasil.

Adicionalmente, como o questionário foi divulgado por múltiplos canais abertos, não foi possível determinar o número total de profissionais de saúde alcançados nem calcular a taxa de resposta, limitando a avaliação da representatividade da amostra. A sub-representação das regiões Norte e Nordeste, a predominância de respondentes com mais de 20 anos de experiência profissional e a ausência de informações sobre a principal modalidade de diálise na qual os participantes atuavam podem limitar ainda mais a generalização dos achados.

Além disso, o questionário foi submetido à revisão qualitativa e a um estudo piloto, mas não passou por validação psicométrica formal. Por fim, o delineamento exploratório e a ausência de amostragem estratificada impediram análises robustas de subgrupos segundo categoria profissional, região geográfica e modelo de financiamento da assistência à saúde.

Apesar dessas limitações, este estudo representa o primeiro levantamento nacional a avaliar de forma sistemática as práticas e percepções relacionadas à sustentabilidade na nefrologia brasileira, fornecendo informações valiosas sobre os desafios atuais e as oportunidades para o avanço de uma assistência nefrológica mais sustentável.

## CONCLUSÃO

Os resultados evidenciam lacunas importantes entre percepção, conhecimento e implementação de práticas sustentáveis na nefrologia brasileira, destacando a necessidade de estratégias estruturadas que integrem políticas públicas, incentivos institucionais e capacitação profissional. A incorporação da sustentabilidade ao cuidado renal deve ser compreendida não apenas como um imperativo ambiental, mas também como uma oportunidade estratégica para aumentar a eficiência, a resiliência e a equidade do sistema de saúde. Nesse contexto, este estudo oferece subsídios relevantes para o planejamento de políticas e intervenções. Embora os desafios sejam globais, sua superação requer abordagens adaptadas ao contexto do SUS e à diversidade regional brasileira.

## Data Availability

Os dados que sustentam os achados deste estudo estão disponíveis mediante solicitação razoável aos autores correspondentes.

## MATERIAL SUPLEMENTAR

O seguinte material online está disponível para o presente artigo:

Material Suplementar – Questionário sobre Conhecimento e Práticas sobre Sustentabilidade em Nefrologia no Brasil.
